# Editorial: The adaptation strategies of plants to alleviate important environmental stresses

**DOI:** 10.3389/fpls.2023.1307399

**Published:** 2023-10-12

**Authors:** Oksana Sytar, Ashwani Kumar

**Affiliations:** ^1^ Institute of Plant and Environmental Sciences, Slovak University of Agriculture, Nitra, Slovakia; ^2^ Metagenomics and Secretomics Research Laboratory, Department of Botany, University of Allahabad (A Central University), Prayagraj, Uttar Pradesh, India

**Keywords:** adaptation, stresses, abiotic, plant health, crop growth

Agriculture, the backbone of global food security, is constantly challenged by various environmental stresses such as drought, temperature fluctuations, soil pollution, and changing rainfall patterns. Researchers have been exploring innovative strategies and technologies to address these challenges and ensure sustainable crop production (Kumar and Dubey, 2020). Over the years, complex arrays of plant responses to various environmental pressures have been documented. However, a clear mechanistic foundation is often missing from these studies, making it difficult to consider them as the basis for plant development and tolerance pathways (**Figure 1**). Our Research topic comprises 13 original research articles contributed by more than 80 authors (total downloads: 3272; total views: 15k). With this Research topic, we aimed to covers various stress responses, precipitation variability, biochar applications, metal toxicity, alleviation of terminal drought stress and various agricultural practices that help the plants to adapt and mitigate different environmental stresses.

**Figure 1:**
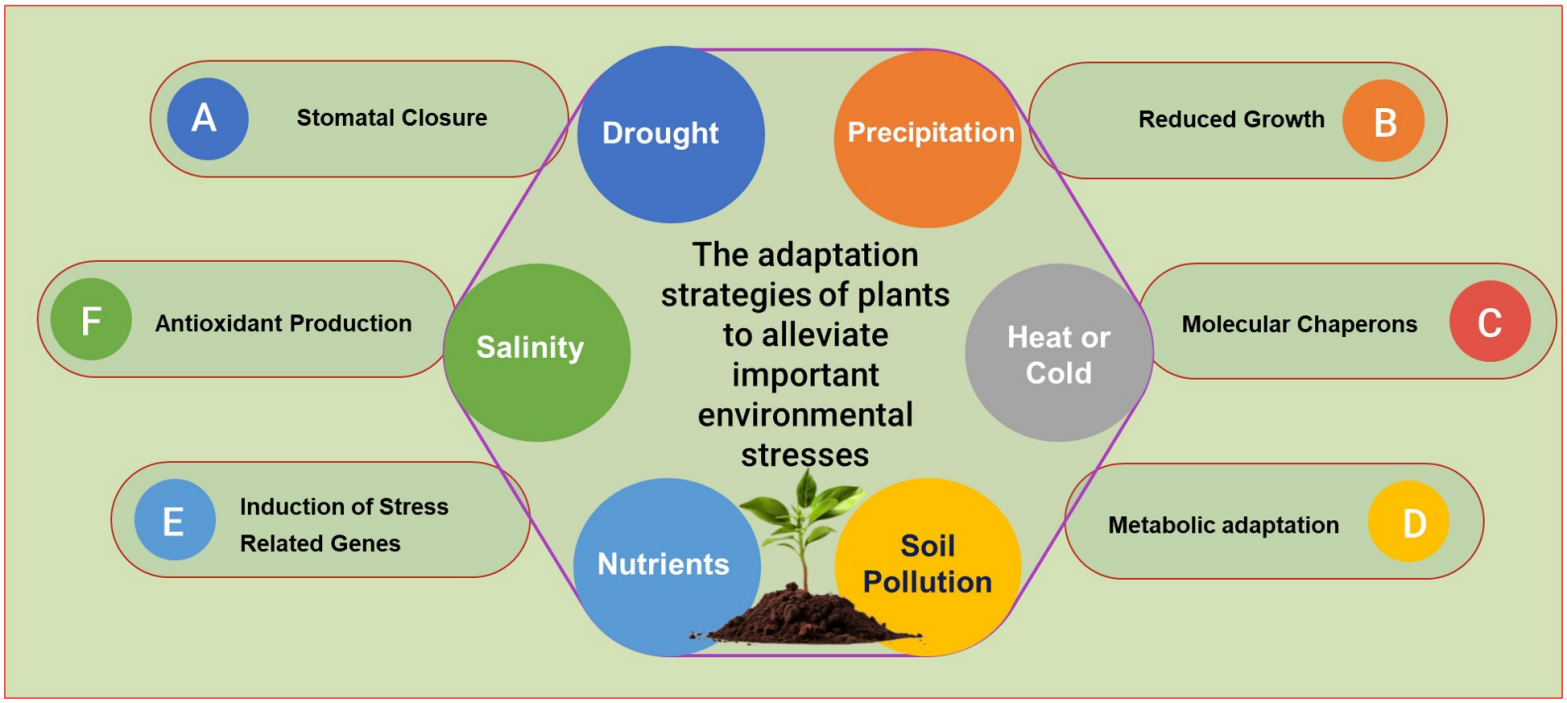
Common plant adaptation strategies against environmental stresses.

Drought stress severely impacts cereal crop growth and yields, making it a top concern for farmers worldwide. The paper titled “*Foliar application of putrescine alleviates terminal drought stress in wheat*” by Wasaya et al. explores the use of putrescine as a potential solution. The research reveals that foliar application of putrescine at a concentration of 1.0 PPM significantly improved wheat’s water status, membrane stability, and yield-related traits under terminal drought stress. This finding offers a promising approach to mitigate drought-related yield losses in wheat cultivation. In water-scarce regions, efficient irrigation methods are essential for cotton cultivation. A study of Singh et al. compares sub-surface drip fertigation (SSDF) with traditional irrigation methods in India. The research highlights the potential of SSDF to save irrigation water, enhance cotton productivity, and improve farmers’ returns, offering a water-savvy concept for sustainable cotton production.

Understanding historical precipitation patterns is crucial for crop management in regions with variable precipitation. The work conducted by Fang et al. on characteristics of historical precipitation for winter wheat cropping analyzes two decades of climate data in a semi-arid and semi-humid area of China. The study characterizes rainfall distribution during key growth stages of winter wheat, providing valuable insights for managing waterlogging and drought risks. In their group, Qin et al. investigated the water use strategies of *Ferula bungeana* in the Badain Jaran Desert. The study reveals that the plant adapts to extreme drought conditions by adjusting its water absorption sources, water potential, hydraulic conductivity, and water use efficiency. Water availability plays a significant role in regulating these strategies. The investigation of Veenstra et al. explores the role of tillering in stabilizing corn yield components under sub-optimal plant densities. It suggests that optimizing plant density to maximize the main stalk primary ears is desirable for maximizing yields. However, tiller expression offers an avenue to reduce dependence on variable optimum plant densities. It suggests that optimizing plant density to maximize the main stalk primary ears is desirable for maximizing yields, but tiller expression offers an avenue to reduce dependence on variable optimum plant densities.

Among environmental stresses, heavy metals are of significant interest in the research related to the impact on plant growth, quality and mitigation of the hard impact of this stress together with the adaptation potential of plants. For example, Wang et al. investigate low-level cadmium exposure in peppermint, this research identifies genes and pathways associated with hormetic responses. Low-level cadmium exposure consistently enhances antioxidant activity in peppermint plants, aiding in their resistance to oxidative damage. This hormetic effect could have implications for peppermint cultivation in contaminated environments.

The study by Abeed et al. titled “*Mitigating cadmium stress in sunflower with microorganisms*” explores the use of *Trichoderma harzianum* and plant growth-promoting bacteria to alleviate cadmium (Cd) stress in sunflower plants. These microorganisms play a vital role in helping sunflower plants adapt to Cd stress by regulating various physiological and biochemical processes, reducing stress markers, and improving water use efficiency. This approach holds promise for enhancing sunflower resilience against Cd-induced damage. Research by Chen into how zinc (Zn) alleviates cadmium (Cd) toxicity in mangrove plants reveals that low-dose Zn treatment positively influences biomass, phenolic acid metabolism-related enzymes, antioxidant capacity, and chlorophyll content. These effects help mitigate Cd toxicity in mangrove plants, indicating a potential strategy for managing heavy metal stress.

Biochar, derived from biomass, has attracted attention for its potential to enhance soil fertility and crop productivity. The study by Wan et al. explores the impact of biochar, specifically wheat-straw biochar, on maize plants under reduced irrigation. Biochar addition significantly increases root parameters, soil nutrient availability, and plant biomass. It suggests combining wheat-straw biochar with partial root-zone drying can improve maize growth and nutrient uptake, particularly in water-limited conditions. Another biochar study by Farhangi-Abriz and Ghasemmi-Golezani examines the impact of chemically modified biochars on mint plants exposed to fluoride and cadmium toxicities. Modified biochars effectively reduce fluoride and cadmium levels in plant leaves, improve soil properties, and enhance plant growth and nutrient uptake. They are more efficient at protecting plants from soil pollutants than solid biochar.

Soybean, widely used in intercropping systems, must adapt to varying light conditions. Wu et al. explore how soybean seedlings adjust to shade. The study reveals that soybean seedlings adapt by altering morphological and physiological traits, highlighting the plant’s plasticity in response to environmental stressors.

The research findings reported in this Research Topic offer promising solutions for mitigating issues such as drought, heavy metal exposure, and variable precipitation patterns. Strategies include the uses of putrescine for drought-tolerant wheat, water-efficient cotton cultivation methods, and optimizing plant density for corn yields. Additionally, studies highlight plant adaptability in response to environmental stressors and the potential of microorganisms and biochar to combat heavy metal stress and enhance soil fertility. These findings hold significant promise for sustainable and resilient agriculture.

## Author contributions

OS: Writing – original draft. AK: Writing – original draft, Writing – review & editing.
